# Integrated multi-omics analysis uncovers key metabolic and transcriptional regulatory networks in *Blumea balsamifera* responding to salt stress

**DOI:** 10.3389/fpls.2026.1766642

**Published:** 2026-03-03

**Authors:** Changmao Guo, Zejun Mo, Su Chen, Kailang Mu, Minghui Huang, Yuan Yuan, Qiumei Luo, Yongfang Wang, Dandan Zhao, Yuchen Liu, Yuxin Pang

**Affiliations:** 1 College of Pharmaceutical Sciences, Guizhou University of Traditional Chinese Medicine, Guiyang, China; 2 School of Biosciences and Biopharmaceutics, Guangdong Pharmaceutical University, Guangzhou, China

**Keywords:** *Blumea balsamifera* (L.) DC., medicinal plant, metabolomics, salt tolerance mechanism, transcriptomics

## Abstract

**Introduction:**

Soil salinization is a key limiting factor for global agricultural production and plant growth. However, the salt tolerance response mechanism of the medicinal plant *Blumea balsamifera* (L.) DC. has not been systematically investigated.

**Methods:**

Fivemonthold seedlings of *B. balsamifera* were used as experimental materials, and five salt treatments were designed: control (CK), low salt (LS), moderate salt (MS), high salt (HS), and extremely high salt (EHS). Growth, photosynthetic, and physiological indices were measured. According to physiological changes, the HS and EHS groups at 12 d of treatment (when plants entered the core stress response stage) were selected for integrated multiomics analysis.

**Results:**

With increasing salt stress, the net photosynthetic rate (Pn), transpiration rate (Tr), and stomatal conductance (Gs) of *B. balsamifera* decreased continuously. The activities of superoxide dismutase (SOD) and catalase (CAT) increased first and then decreased, synergistically removing reactive oxygen species (ROS) with peroxidase (POD). Changes in osmotic adjustment substances and elevated lignin (LIG) content implied enhanced cell wall–related processes. Metabolomic analysis identified 677 and 692 differentially accumulated metabolites (DAMs) in HS vs CK and EHS vs CK, respectively, both enriched in flavone and flavonol biosynthesis. Transcriptomic analysis detected 30,213 and 13,644 differentially expressed genes (DEGs) in HS vs CK and EHS vs CK, respectively, both enriched in oxidative phosphorylation. Integrated analysis demonstrated that oxidative phosphorylation, flavone and flavonol biosynthesis, and cutin, suberine, and wax biosynthesis were the core response pathways, which mediated salt tolerance by regulating key DAMs (e.g., fumaric acid, kaempferol3Orutinoside, luteolin) and DEGs (e.g., flavonoid 3’monooxygenase, peroxygenaselike isoform X2).

**Discussion:**

This study systematically clarifies the salt tolerance mechanism of *B. balsamifera*, providing a theoretical basis for its salttolerant breeding and the utilization of medicinal plant resources in salinized regions.

## Introduction

Soil salinization is one of the major challenges confronting global agriculture and ecological environments, currently affecting approximately 20% of irrigated farmlands worldwide (Li et al., 2021b; Sarri et al., 2021). High concentrations of water-soluble salts such as Na^+^ and Cl^−^ in salinized soils induce osmotic imbalance, ionic toxicity, and oxidative stress in plants, inhibiting photosynthesis, disrupting metabolic homeostasis, inducing leaf chlorosis and reduced plant height, and ultimately leading to growth retardation or even death (Yang and Guo, 2018; Zhu et al., 2025). Medicinal plants constitute crucial sources of natural medicines, and their growing habitats are constantly threatened by soil salinization in marginal lands (Liu et al., 2022a). Current studies have confirmed that abiotic stress can regulate the accumulation patterns of bioactive components in medicinal plants, and certain stress conditions can also significantly increase the contents of multiple medicinal ingredients (Shen et al., 2020; Shi et al., 2023). Therefore, elucidating the physiological and molecular mechanisms underlying the salt stress response of medicinal plants holds significant theoretical and practical importance for the breeding of salt-tolerant varieties and standardized cultivation.

*Blumea balsamifera* (L.) DC., a perennial herbaceous plant belonging to the genus Blumea of the Asteraceae family, is natively distributed in Southwest China. Its dried leaves can be processed to extract blumea camphor and L-borneol, making it a valuable traditional Chinese medicinal herb with effects such as inducing resuscitation, clearing heat, and relieving pain (Guan et al., 2022; Huang et al., 2025; Putra et al., 2024). Additionally, *B. balsamifera* is rich in bioactive components, including flavonoids and terpenoids, possessing broad application value in pharmaceuticals, cosmetics, and other fields (Zhang et al., 2021a). Currently, research on *B. balsamifera* has mainly focused on resource investigation, cultivation technology optimization, and bioactive component extraction, whereas its stress adaptability, especially the mechanism underlying salt stress response, has not been systematically reported. Previous studies have verified that the salt stress response of Asteraceae plants presents both interspecific commonality and species-specific characteristics, and most species of this family resist salt injury by regulating the antioxidant system, ABA signaling pathway and other biological processes (Xia et al., 2025). However, the phenotypic plasticity, physiological and metabolic remodeling, and molecular regulatory networks of *B. balsamifera* under salt stress remain unknown.

The salt stress response in plants is a complex process involving coordinated regulation across multiple levels and pathways (Khatun et al., 2021). Adaptive alterations in plant phenotype and photosynthetic system, together with the dynamic homeostasis of antioxidant enzymes and osmotic adjustment substances, constitute the core physiological foundation for plants to resist oxidative damage and osmotic stress (Liu et al., 2022b; Ma et al., 2025). In recent years, metabolomic and transcriptomic analyses have become powerful technical tools for elucidating the molecular mechanisms of plant stress responses. These approaches have been widely applied in salt-tolerance studies of diverse plant species, including *Glycyrrhiza uralensis* Fisch (Lv et al., 2024), *Solanum lycopersicum* L (Li et al., 2024). and *Lycium barbarum* L (Qin et al., 2022), providing critical technical support for elucidating the synergistic regulatory mechanism between genes and metabolites under stress conditions. However, studies on the dynamic changes of physiological indices and multi-omics correlation analysis of *B. balsamifera* under salt stress have not yet been conducted, and the synergistic mechanism of key regulatory genes and differential metabolites remains to be elucidated.

Based on this, the present study used seedlings of *B. balsamifera* as materials, set up gradient salt concentration treatments, and dynamically determined phenotypic growth, photosynthetic characteristics, and leaf physiological and biochemical indices of *B. balsamifera* under salt stress. Combined with integrated metabolomic and transcriptomic analyses, we screened Differentially Accumulated Metabolites (DAMs) and Differentially Expressed Genes (DEGs) in response to salt stress, identified key response time points, and elucidated core regulatory pathways and molecular regulatory networks. This study aims to clarify the physiological and molecular mechanisms underlying salt stress adaptation in *B. balsamifera*; it not only enriches salt tolerance gene resources of medicinal plants but also provides theoretical support and practical reference for the breeding of salt-tolerant *B. balsamifer*a varieties, the development of medicinal resources on salinized lands, and standardized cultivation.

## Materials and methods

### Plant materials and salt stress treatment

Seeds of *B. balsamifera* were collected in Duanqiao Town, Guanling County, Anshun City, Guizhou Province, China (N 19°30’36’’, E 109°34’12’’) in April 2024. Identified by Professor Yuxin Pang from Guizhou University of Traditional Chinese Medicine in accordance with the classification criteria of *Flora Reipublicae Popularis Sinicae*, the seeds were confirmed to belong to *B. balsamifera* (Asteraceae). Subsequently, sowing and seedling raising were conducted in No. 2 Greenhouse of the Medicinal Plant Cultivation Experimental Nursery at Guizhou University of Traditional Chinese Medicine (N 26°22’20’’, E 106°27’34’’), where seedlings were grown in a non−thermostatic greenhouse with natural ventilation and daylight transmission. The seedling substrate adopted a general−purpose formula manufactured by Xiangzheng Agricultural Technology, which was compounded from peat, coco coir, vermiculite and perlite; plastic pots with a height of 85 mm and a diameter of 100 mm were used as cultivation containers, with one seedling planted per pot. In September 2024, 5-month-old uniform seedlings (average plant height: 11.67 cm) were selected as experimental materials for salt stress-related studies.

Based on preliminary experiment results (100 mmol·L^-1^ NaCl treatment for 2 weeks resulted in good plant growth without significant mortality), this experiment adopted a completely randomized design with five NaCl concentration gradients, consisting of 0mmol·L^-1^ (control, CK), 50 mmol·L^-1^ (Low Salt, LS), 100 mmol·L^-1^ (Medium Salt, MS), 150 mmol·L^-1^ (High Salt, HS) and 200 mmol·L^-1^ (Extra High Salt, EHS). The 150 screened seedlings were randomly allocated to five treatment groups with 30 plants per group, and three biological replicates (n = 3) were arranged for each treatment (Manaa et al., 2011; Rajabi Dehnavi et al., 2024). During the experiment, 100 mL of NaCl solution with the corresponding concentration was irrigated to each treatment group between 7:00 and 8:00 AM every 3 days, ensuring leachate from the bottom of pots to maintain uniform salt concentration in the growth medium. On sampling days (Days 6, 12, 18, and 24), sample collection was performed prior to irrigation, while the same irrigation protocol was followed on non-sampling days. Samples were collected on Days 0, 6, 12, 18, and 24 after experiment initiation. Three plants were randomly selected per treatment, and growth parameters and photosynthetic traits were measured first. Subsequently, the 3rd to 5th functional leaves from the middle part of each plant were selected, and leaf tissues were sampled using a sterile punch, avoiding the midrib. A portion of fresh leaves was immediately used for the determination of relative water content (RWC), while the remaining tissues were quickly placed into labeled sterile cryovials, snap-frozen in liquid nitrogen, and stored at -80°C for subsequent analyses of physiological and biochemical indices as well as transcriptomic and metabolomic profiling.

### Determination of growth parameters

Plant height (PH), leaf length (LL), and leaf width (LW) were measured using a tape measure. Leaf thickness (LT) and basal stem diameter (BS, defined as the stem diameter parallel to the ground near the soil surface) were determined with a vernier caliper. Leaf relative water content (RWC) was measured via the saturated weight method (Ghazouani et al., 2023), using the following formula:


$$RWC=\frac{\left(FW-DW\right)}{\left(TW-DW\right)}\times 100\%$$

Where: FW = fresh weight of leaves; TW = turgid weight of leaves; DW = dry weight of leaves.

### Determination of photosynthetic parameters

Photosynthetic-related parameters were measured using a portable LCI photosynthesis system (Beijing AoZuo Ecological Instruments Co., Ltd.) prior to sampling, conducted between 9:00-11:00 AM under a fixed photosynthetic photon flux density of 800 μmol m^-2^s^-1^. The measured parameters included net photosynthetic rate (Pn), stomatal conductance (Gs), transpiration rate (Tr), and intercellular CO_2_ concentration (Ci).

### Determination of physiological and biochemical indices

All physiological indices were determined using the microplate method with commercial kits produced by Grace Biotechnology Co., Ltd. (Suzhou, China), and the specific detection procedures are as follows: Malondialdehyde (MDA) content was measured via the thiobarbituric acid colorimetric method, and calculated based on the absorbance difference between 532 nm and 600 nm after the condensation reaction with thiobarbituric acid. Superoxide dismutase (SOD) activity was assayed using the WST-8 method, with absorbance detected at 450 nm, and enzyme activity was quantified according to its inhibitory capacity on WST-8 reduction. Catalase (CAT) activity was determined by ultraviolet spectrophotometry, and the CAT activity in samples was calculated on the basis of the decrease in hydrogen peroxide content monitored at 510 nm. Peroxidase (POD) activity was detected with the guaiacol chromogenic method, and enzyme activity was computed by detecting the variation in absorbance at 470 nm during the oxidation of guaiacol by H_2_O_2_. Lignin (LIG) content was measured using the acetylation method, and absorbance was determined at 280 nm after the reaction for subsequent content calculation. Soluble sugar (SS) content was quantified via the anthrone colorimetric method, and calculated by measuring absorbance at 620 nm following the dehydration-condensation reaction with anthrone-sulfuric acid reagent. Soluble protein (SP) content was assayed using the Coomassie Brilliant Blue G-250 method; relying on the characteristic that G-250 binds to proteins to form a blue complex, its content was calculated by detecting absorbance at 600 nm. Proline (PRO) content was determined by the ninhydrin colorimetric method, and after the salicylic acid extract reacted with acidic ninhydrin in a boiling water bath, absorbance was measured at 520 nm to calculate the final content.

### Determination of key sampling time points for metabolomic and transcriptomic analyses

Comprehensive analysis of physiological indices in *B. balsamifera* leaves revealed that, compared with CK, significant physiological differences in most indices under LS and MS treatments emerged as late as Day 12 or even Day 18 of treatment. In contrast, HS and EHS treatments exhibited significant differences from CK as early as Day 6, and most key physiological indices (e.g., CAT, POD, SOD activities, and SP content) peaked on Day 12, indicating that plants had entered a critical transition stage from stress adaptation to salt response at this time point. Therefore, samples from CK, HS, and EHS treatments on Day 12 were selected for metabolomic and transcriptomic sequencing analyses, aiming to elucidate the expression differences and regulatory mechanisms of key genes involved in the salt stress response of *B. balsamifera*.

### Metabolomic analysis

A UHPLC-Q Exactive HF-X mass spectrometer (Thermo Fisher Scientific, USA) and an HSS T3 column (100 mm × 2.1 mm i.d., 1.8 µm; Waters Corporation, USA) were used in this experiment. Metabolite extraction and derivatization followed the method described by Han et al (Han et al., 2024), while gas chromatography conditions were referenced from the study by Ren et al (Ren et al., 2022). Raw data were subjected to baseline filtering, peak detection, integration, and retention time correction using Progenesis QI (Waters Corporation, Milford, USA). Metabolite information was acquired by matching against the HMDB (http://www.hmdb.ca/), Metlin (https://metlin.scripps.edu/), and Meiji in-house database. The data matrix was processed as follows: missing values were removed based on the 80% rule, imputed with the minimum value, normalized by total sum scaling, variables with relative standard deviation (RSD) > 30% in quality control (QC) samples were eliminated, and a log10 transformation was performed. Principal component analysis (PCA) and orthogonal partial least squares-discriminant analysis (OPLS-DA) were conducted using the ropls package (Version 1.6.2) in R software with 7-fold cross-validation. Significantly differential metabolites were screened with VIP > 1 and p< 0.05 (Liu et al., 2023). Metabolic pathway annotation of differential metabolites was performed using the KEGG database (https://www.kegg.jp/kegg/pathway.html) to identify the pathways involved.

### Transcriptomic analysis

Total RNA was extracted from 9 samples using the MJZol total RNA extraction kit (Shanghai Meiji Biomedical Technology Co., Ltd.). RNA concentration and purity were determined with a Nanodrop 2000, integrity was assessed by agarose gel electrophoresis, and the RNA Quality Number (RQN) was measured using an Agilent 5300. After initial quality control and filtering of raw paired-end sequencing reads, trimming and further quality control were performed with the fastp tool based on the following criteria: removal of reads containing adapter sequences, reads with an unknown base (N) ratio exceeding 5%, and reads where the proportion of bases with a quality score ≤ 20 exceeded 20%. The resulting high-quality clean data were used for subsequent analyses. In the deduplication, filtering, and optimization pipeline, all clean data were first subjected to *de novo* assembly using Trinity software to generate initial Unigenes. Subsequently, the CD-HIT program was used for clustering and deduplication of initial Unigene sequences, with a sequence similarity threshold set at 0.95, and sequences shorter than 200 bp were filtered out to obtain non-redundant Unigenes. Differential gene expression analysis was performed using DESeq2 software, with screening thresholds set as |log2 fold change| ≥ 1 and padjust< 0.05 (Oh et al., 2023). Gene expression pattern clustering was conducted based on gene expression profiles across different samples. Furthermore, functional annotation and enrichment analysis of Differentially Expressed Genes (DEGs) in the gene set were performed using the Gene Ontology (GO, http://www.geneontology.org/) and Kyoto Encyclopedia of Genes and Genomes (KEGG, http://www.genome.jp/kegg/) databases. The raw transcriptomic sequencing data of this study have been successfully submitted to the NCBI Sequence Read Archive (SRA) database, with the accession number SRP649295.

### Integrated correlation analysis of metabolomic and transcriptomic data

To elucidate the potential transcriptional regulatory mechanisms underlying the salt-tolerant metabolic pathways of *B. balsamifera*, Pearson correlation analysis was performed to explore the association between metabolomic and transcriptomic datasets. For the HS vs CK and EHS vs CK comparison groups, correlations between the top 20 Differentially Accumulated Metabolites (DAMs) and Differentially Expressed Genes (DEGs) were analyzed, with thresholds set as P-value ≥ 1 and the absolute value of the Pearson correlation coefficient (| rho |) ≤ 0 to filter out irrelevant DAMs or DEGs, with | rho | > 0.8 and *P* < 0.05 as the criteria for screening target DEGs and DAMs (Li et al., 2021a). The analysis was conducted using scipy (Python 1.0.0), and correlation heatmaps were generated. Furthermore, to identify the key KEGG pathways involved in the salt stress response of *B. balsamifera* leaves, key enriched pathways from both transcriptomic and metabolomic analyses—including Oxidative phosphorylation, Flavone and flavonol biosynthesis, and Cutin, suberine and wax biosynthesis—were prioritized for in-depth interpretation, along with their associated genes and metabolites.

### Quantitative real-time PCR

To verify the accuracy of differential mRNA expression results identified by high-throughput sequencing (RNA-Seq), nine genes from salt stress-related pathways—including Ribosome, Oxidative phosphorylation, Cutin, suberine and wax biosynthesis, and Flavone and flavonol biosynthesis—were selected for qRT-PCR validation. Their primer sequences and functional annotations are provided in **Supplementary Table 1**. Total RNA was extracted using the MJZol total RNA extraction kit (Shanghai Meiji Biotechnology, China), and cDNA was synthesized via reverse transcription with ExonScript RT Mix (containing dsDNase). The 18S rRNA gene was used as the reference gene (primer sequences: F: CGGCTACCACATCCAAGGAA; R: GCTGGAATTACCGCGGCT). qPCR reactions were performed strictly following the manufacturer’s instructions for the SYBR PRIME qPCR Kit (Fast HS, Cat. No. BG0014). Three transcriptomic samples were selected at each time point, with three technical replicates per biological replicate. The relative expression levels of target genes were calculated using the 2^−△△Ct^ method and compared with the RNA-Seq results.

### Data statistics and analysis

Statistical analyses were performed using SPSS 22.0 and Excel 2021, while graphs were constructed with GraphPad Prism 9.5 and OriginPro 2024. All data are presented as the mean ± standard deviation (SD) from three replicates. Duncan’s new multiple range test was used to determine significant differences, with different lowercase letters indicating significant differences among different treatments at the same time point (*P* < 0.05).

## Results

### Phenotypic responses

**Figure 1:**
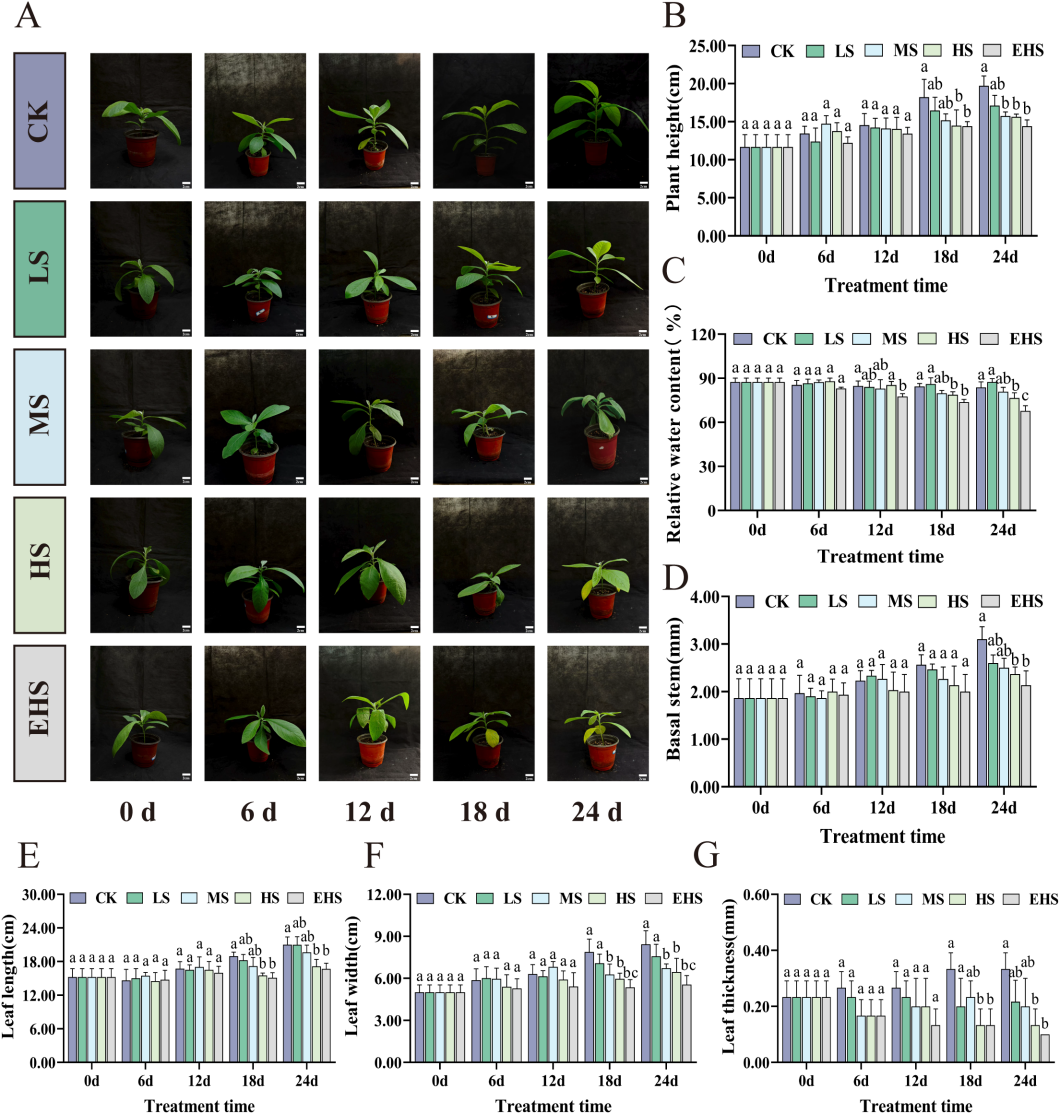
Effects of salt stress on the growth morphology of *B. balsamifera.***(A)** Phenotypic responses. **(B-G)** Comparisons of plant height (PH), relative water content (RWC), basal stem (BS), leaf length (LL), leaf width (LW), and leaf thickness (LT) under different treatments. Different lowercase letters in the figure indicate significant differences (*P<* 0.05), while the same lowercase letters indicate no significant differences (*P* > 0.05).

With increasing salt concentration and prolonged treatment duration, phenotypic damage of *B. balsamifera* gradually intensified. At the late stage of treatment, plants under HS and EHS stress exhibited significant wilting accompanied by leaf chlorosis, while those under CK and LS treatments remained relatively normal (**Figure 1A**). PH in the CK group continued to increase over time, whereas growth was significantly inhibited in salt-stressed groups at 18 and 24 days (**Figure 1B**). RWC, the CK group maintained a high level, while RWC in salt-stressed groups showed a decreasing trend with increasing salt concentration and treatment duration. At 24 days, RWC in HS and EHS groups reached the lowest values, decreasing by 8.55% and 19.09% compared with the CK group, respectively, indicating that high salt stress caused severe leaf water deficit (**Figure 1C**). For BS, HS and EHS treatments resulted in significantly lower values than the CK group at 24 days (**Figure 1D**). In terms of leaf morphological parameters (LL, LW, LT), growth was inhibited with increasing salt concentration, and HS/EHS groups were significantly lower than the CK group at both 18 and 24 days (**Figures 1E-G**). Overall, the inhibitory effect of salt stress on the growth and morphology of *B. balsamifera* was concentration- and time-dependent. Under HS and EHS stress, plant growth, water status, and leaf morphological parameters were all subjected to significant negative impacts.

### Photosynthetic responses

**Figure 2:**
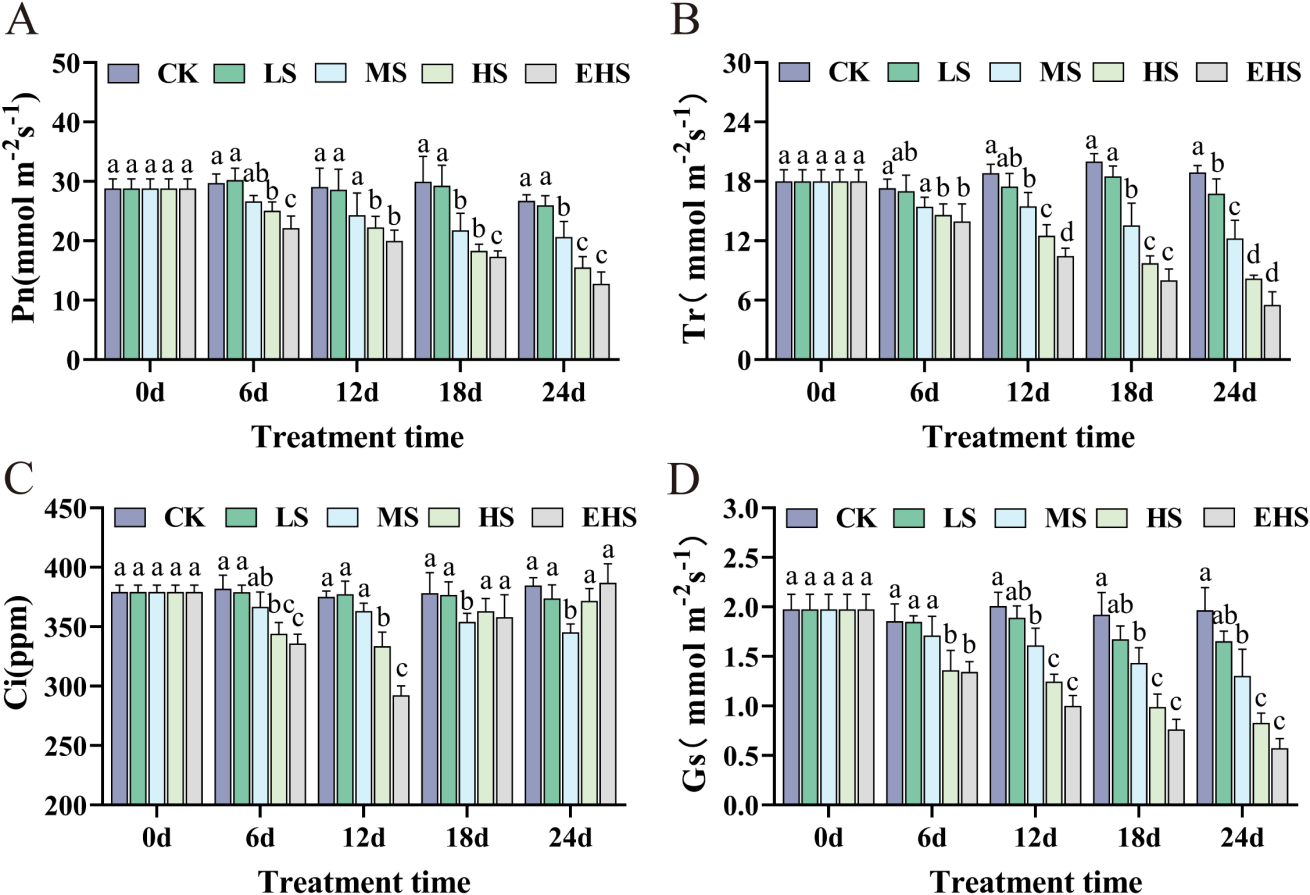
Effects of salt stress on photosynthesis-related parameters of *B. balsamifera*. **(A)** Net photosynthetic rate (Pn). **(B)** Transpiration rate (Tr). **(C)** Intercellular CO_2_ concentration (Ci). **(D)** Stomatal conductance (Gs). Different lowercase letters in the figure indicate significant differences (*P* < 0.05), while the same lowercase letters indicate no significant differences (*P* > 0.05).

Under salt stress, there was no significant difference in Pn between the LS treatment and CK group, while Pn in MS, HS, and EHS treatments decreased significantly with prolonged stress duration. For instance, at 24 days, Pn in the EHS treatment decreased by approximately 52.28% compared with CK, and the inhibitory effect became more pronounced with increasing salt concentration and stress duration (**Figure 2A**). The variation trend of Tr was similar to that of Pn. No significant difference in Tr was observed between LS and CK during the first 18 days; however, at 24 days, Tr in the EHS treatment was reduced by approximately 70.74% relative to CK, indicating that high salt stress exerted a significant inhibitory effect on water transpiration (**Figure 2B**). Ci showed no significant difference between LS and CK, while it fluctuated in HS and EHS treatments—decreasing to the lowest at 12 days and then recovering. This suggests that photosynthetic carbon assimilation may be dominated by non-stomatal limiting factors under high salt stress (**Figure 2C**). Gs in the LS treatment was not significantly different from CK, whereas Gs in MS, HS, and EHS treatments decreased significantly with increasing salt stress intensity and duration. At 24 days, Gs in HS and EHS treatments were reduced by approximately 57.87% and 70.85% compared with CK, respectively, indicating that high salt stress affects leaf gas exchange processes by reducing Gs (**Figure 2D**).

### Physiological and biochemical responses

**Figure 3:**
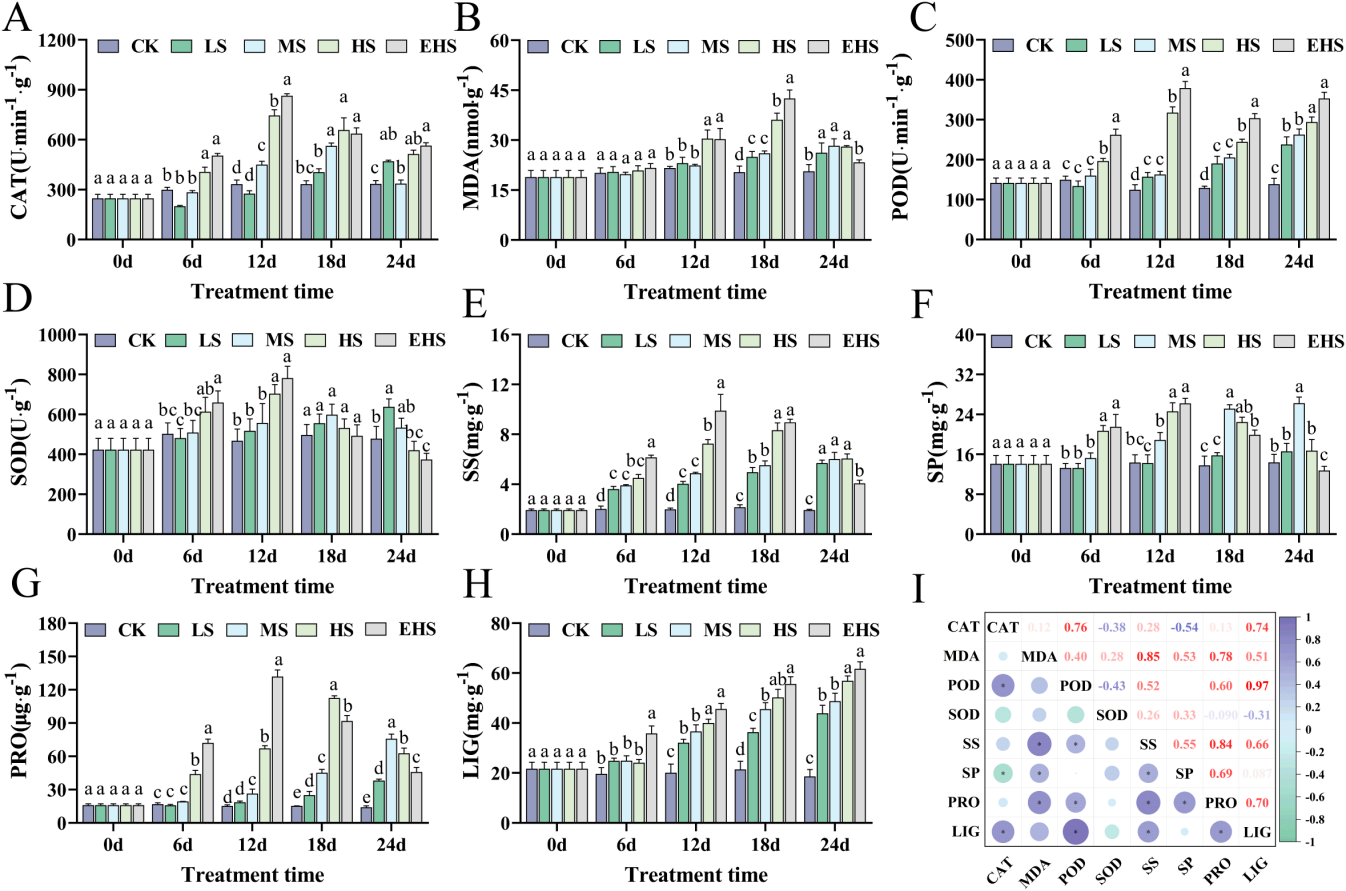
Effects of salt stress on leaf physiological and biochemical indices of *B. balsamifera*. **(A)** Catalase (CAT) activity. **(B)** Malondialdehyde (MDA) content. **(C)** Peroxidase (POD) activity. **(D)** Superoxide dismutase (SOD) activity. **(E)** Soluble sugar (SS) content. **(F)** Soluble protein (SP) content. **(G)** Proline (PRO) content. **(H)** Lignin (LIG) content. **(I)** Heatmap of correlations among physiological indices. Different lowercase letters in the figure indicate significant differences (*P* < 0.05), while the same lowercase letters indicate no significant differences (*P* > 0.05).

Under salt stress, physiological and biochemical indices of *B. balsamifera* leaves—including antioxidant enzyme activities (CAT, POD, SOD) and osmoregulatory substances—exhibited significant time- and concentration-dependent changes. CAT activity peaked on Day 12 under HS and EHS treatments, increasing by approximately 124.08% and 159.89% compared with CK, respectively, showing significant differences among salt gradients and treatment durations (**Figure 3A**). Meanwhile, MDA (a marker of membrane lipid peroxidation) content in the EHS treatment increased by 108.51% relative to CK on Day 18, indicating intensified membrane damage with increasing stress intensity and duration (**Figure 3B**). POD, SOD activities, and SP content followed a similar trend to CAT, peaking on Day 12 under HS and EHS treatments before declining, suggesting rapid activation of the leaf antioxidant system and protein metabolism to scavenge reactive oxygen species (ROS) and maintain cellular homeostasis (**Figures 3C, D, F**). SS content remained significantly higher in all salt-treated groups than in CK on Day 24, providing a foundation for maintaining cellular homeostasis through osmotic regulation (**Figure 3E**). Notably, PRO content reached a peak on Day 12 under EHS treatment (approximately 8.66 times higher than that in CK), confirming it as a key osmoregulatory substance in response to salt stress (**Figure 3G**). Additionally, plants enhanced cell wall rigidity by increasing LIG content to adapt to salt stress (**Figure 3H**). Furthermore, a correlation heatmap revealed tight associations among indices: for example, POD activity was strongly positively correlated with LIG content (r = 0.97), and SS content was positively correlated with MDA content (r = 0.85), highlighting the synergistic effects and strong interconnections between physiological indices (**Figure 3I**).

### Metabolomic responses

**Figure 4:**
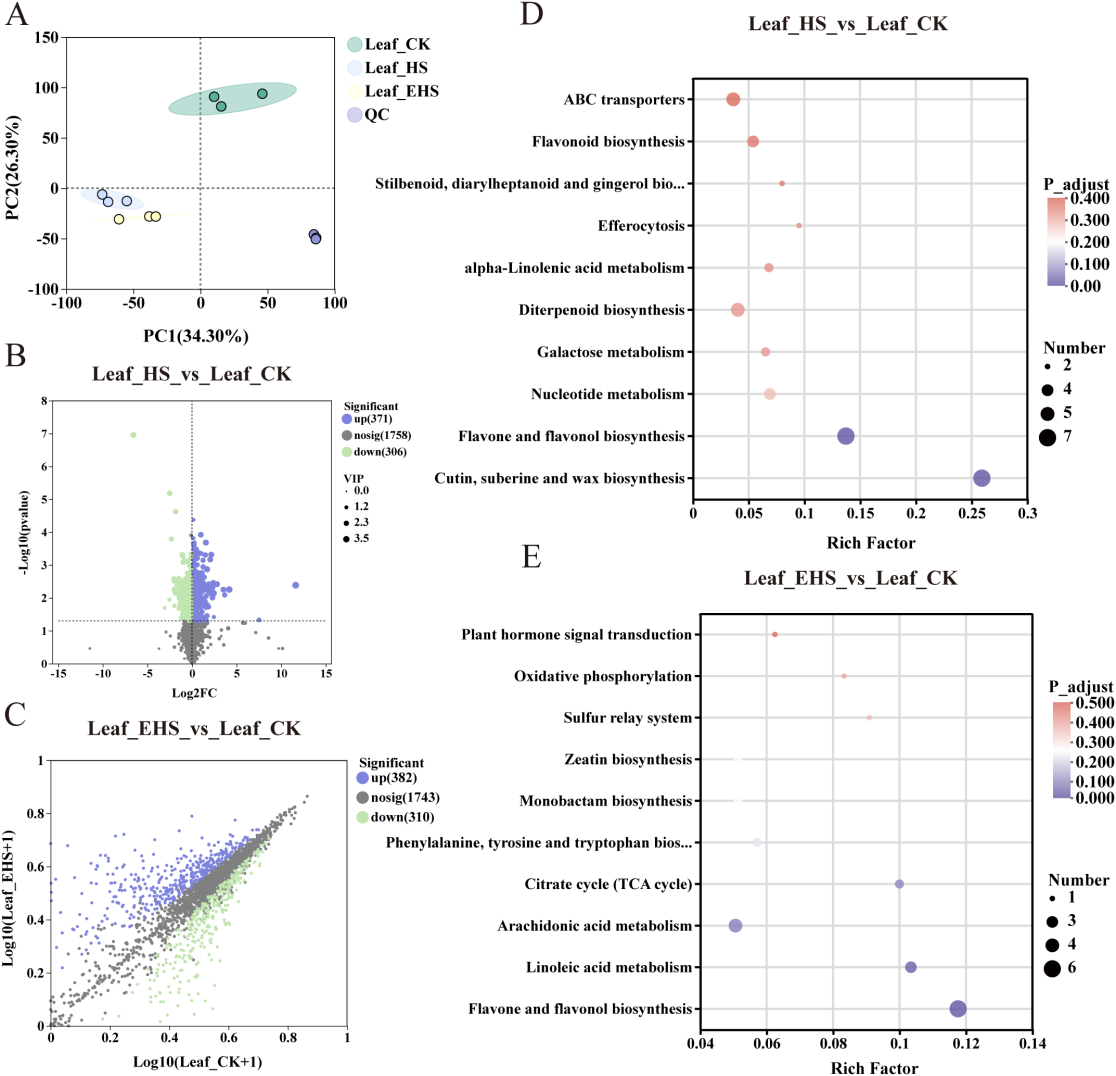
Metabolome expression analysis. **(A)** Principal component analysis: Each point represents a biological replicate in the grouped experiment, with different colors representing different groups. **(B, C)** Volcano plots of Differential Accumulation Metabolites (DAMs): They show the distribution of DAMs for the comparisons HS vs. CK and EHS vs. CK, respectively. Purple dots represent significantly upregulated DAMs, green dots represent significantly downregulated DAMs, and gray dots represent metabolites with no significant differences. **(D, E)** KEGG pathway enrichment analysis: They show the KEGG pathway enrichment results of DAMs for the comparisons HS vs. CK and EHS vs. CK, respectively.

Based on untargeted metabolomic analysis, there was distinct separation among samples under different salt treatments, indicating that metabolite accumulation at the metabolomic level responded well to salt stress intensity (**Figure 4A**). When comparing HS and EHS groups with CK separately, a total of 677 Differentially Accumulated Metabolites (DAMs) were identified in the HS vs CK comparison, among which 371 were upregulated and 306 were downregulated; in the EHS vs CK comparison, 692 DAMs were obtained, with 382 upregulated and 310 downregulated (**Figures 4B, C**). KEGG pathway enrichment analysis revealed that DAMs in the HS vs CK group were significantly enriched in pathways including Cutin, suberine and wax biosynthesis, Flavone and flavonol biosynthesis, and Nucleotide metabolism, while DAMs in the EHS vs CK group were significantly enriched in Flavone and flavonol biosynthesis, Linoleic acid metabolism, and Arachidonic acid metabolism (**Figures 4D, E**). Notably, Flavone and flavonol biosynthesis was a pathway significantly enriched with DAMs in both comparisons, suggesting that when *B. balsamifera* responds to HS and EHS stress, it may prioritize regulating the Flavone and flavonol biosynthesis pathway to restructure the secondary metabolic network for adaptation to saline environments; relevant DAMs are listed in **Supplementary Table 2**.

### Transcriptomic responses

**Figure 5:**
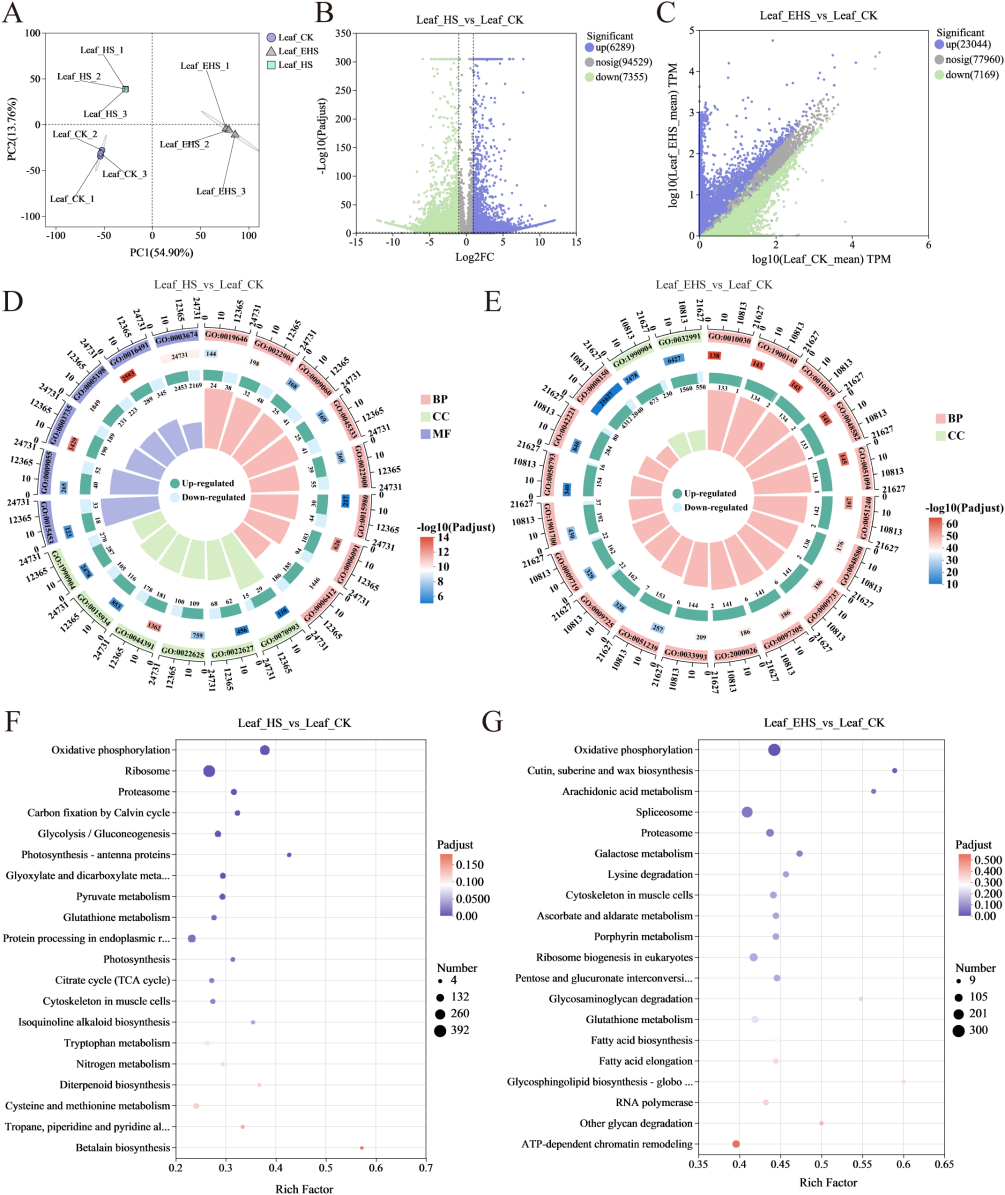
Transcriptome expression analysis. **(A)** Principal component analysis: Each point represents a biological replicate in the grouped experiment, with different colors distinguishing different groups. **(B, C)** Volcano plots of Differentially Expressed Genes (DEGs): They show the distribution of DEGs for the comparisons HS vs. CK and EHS vs. CK, respectively. Purple dots represent significantly upregulated DEGs, green dots represent significantly downregulated DEGs, and gray dots represent genes with no significant differences. **(D, E)** GO functional enrichment circle plots: They present the GO functional enrichment results of DEGs for the comparisons HS vs. CK and EHS vs. CK, respectively. From outside to inside: enrichment classification, number of background genes, statistical significance, proportion of upregulated and downregulated genes, and Rich Factor. **(F, G)** KEGG pathway enrichment analysis: They show the KEGG pathway enrichment results of DEGs for the comparisons HS vs. CK and EHS vs. CK, respectively.

Quality control (QC) results of sequencing data for nine *B. balsamifera* leaf samples are summarized in **Supplementary Table 3**, and detailed comparison data of sequencing and assembly results are provided in 0 4. Principal component analysis (PCA) showed that PC1 and PC2 explained 54.90% and 13.76% of the gene expression variation among samples, respectively (**Figure 5A**). Consistent with the metabolomic analysis results, samples from different salt treatment groups exhibited distinct separation at the transcriptomic level. The EHS vs CK comparison showed the largest number of Differentially Expressed Genes (DEGs), with a total of 30,213 DEGs (23,044 upregulated and 7,169 downregulated), whereas the HS vs CK comparison had fewer DEGs (13,644 in total, with 6,289 upregulated and 7,355 downregulated) (**Figures 5B, C**). The trend in DEG numbers indicated that increased salt concentration may be a key factor driving significant changes at the transcriptomic level.

GO functional enrichment analysis revealed that DEGs in the HS vs CK group were significantly enriched in GO terms related to molecular function, biological process, and cellular component (e.g., oxidoreductase activity, GO:0016491; generation of precursor metabolites and energy, GO:0006091; ribosomal subunit, GO:0044391) (**Figure 5D**). In contrast, DEGs in the EHS vs CK group were mainly enriched in GO terms associated with biological process (e.g., positive regulation of seed germination, GO:0010030; regulation of seedling development, GO:1900140) (**Figure 5E**). KEGG pathway enrichment analysis showed that DEGs in the HS vs CK group were significantly enriched in pathways such as Oxidative phosphorylation, Ribosome, and Proteasome (**Figure 5F**), while those in the EHS vs CK group were significantly enriched in pathways including Oxidative phosphorylation, Cutin, suberine and wax biosynthesis, and Arachidonic acid metabolism (**Figure 5G**).

### Correlation analysis of DAMs and DEGs and KEGG pathway enrichment analysis

**Figure 6:**
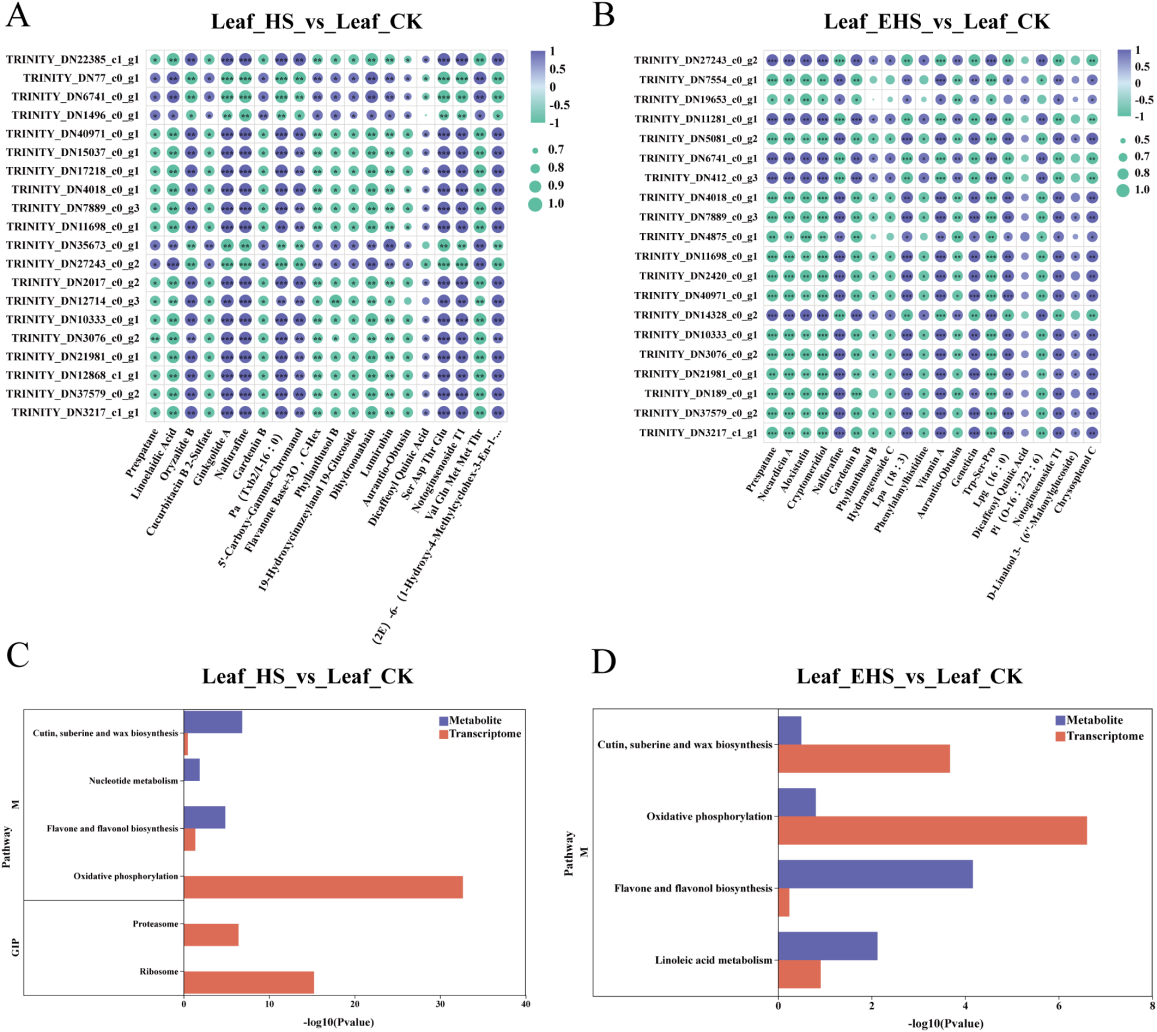
Association analysis between metabolome and transcriptome. **(A)** Correlation analysis of DAMs and DEGs for the comparison HS vs. CK: It shows the correlation between DAMs and DEGs in the HS vs. CK group. **(B)** Correlation analysis of DAMs and DEGs for the comparison EHS vs. CK: It shows the correlation between DAMs and DEGs in the EHS vs. CK group. **(C)** KEGG functional enrichment map of DAMs and DEGs for the comparison HS vs. CK: It presents the KEGG functional enrichment results of DAMs and DEGs in the HS vs. CK group. **(D)** KEGG functional enrichment map of DAMs and DEGs for the comparison EHS vs. CK: It presents the KEGG functional enrichment results of DAMs and DEGs in the EHS vs. CK group.

Under salt stress, the absolute values of | rho | between the top 20 DAMs and DEGs were mostly distributed in the range of 0.8~1.0 for both the HS vs CK and EHS vs CK groups, indicating strong transcription-metabolism coordinated regulatory relationships between these molecular pairs under salt stress. For example, in both groups, the triterpenoid saponin metabolite Notoginsenoside T1 showed a significant positive correlation with most transcription factors, while the flavonoid metabolites Gardenin and BAurantio-Obtusin exhibited a significant negative correlation with most transcription factors (**Figures 6A, B**). KEGG functional enrichment analysis revealed that DAMs and DEGs in the HS vs CK group were enriched in Cutin, suberine and wax biosynthesis and Flavone and flavonol biosynthesis; notably, although no DAMs were enriched in Oxidative phosphorylation, DEGs were significantly enriched in this pathway (**Figure 6C**). In the EHS vs CK group, Oxidative phosphorylation, Cutin, suberine and wax biosynthesis, and Flavone and flavonol biosynthesis were all enriched (**Figure 6D**), demonstrating that these pathways are core regulatory pathways for *B. balsamifera* in response to salt stress, and Oxidative phosphorylation under HS stress was mainly regulated at the transcriptional level. Thus, we focused on analyzing the Oxidative phosphorylation, Flavone and flavonol biosynthesis, and Cutin, suberine and wax biosynthesis pathways, as well as their associated genes and metabolites.

**Figure 7:**
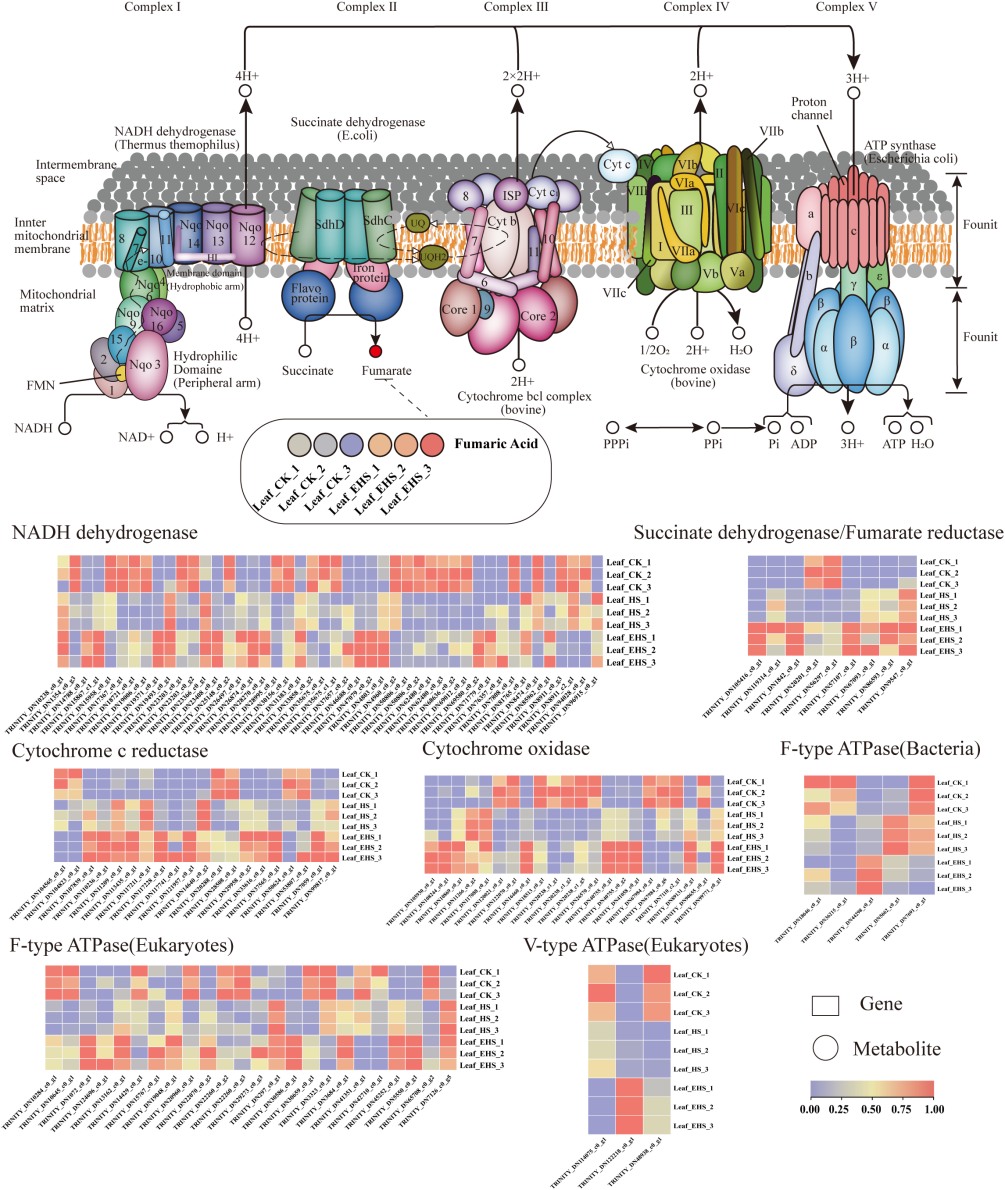
Expression of DAMs and DEGs related to the oxidative phosphorylation pathway. The schematic diagram of the mitochondrial oxidative phosphorylation pathway shows the localization of complexes I-V on the inner mitochondrial membrane, the electron transport chain, and proton transmembrane transport. For the expression of related molecules: in the heat map, boxes represent genes, circles represent metabolites, and the color gradient from purple to red corresponds to the expression levels of the molecules (purple indicates low expression, and red indicates high expression).

Oxidative phosphorylation, the core pathway of mitochondrial energy metabolism, undergoes functional remodeling as a key adaptive strategy for plants in response to salt stress. This pathway involves the spatial arrangement of Complexes Ⅰ~Ⅴ in the inner mitochondrial membrane, electron transfer through the electron transport chain (ETC), and the formation of a transmembrane proton gradient. Heatmap analysis revealed that most genes in this pathway were significantly differentially expressed in salt-stressed groups (HS, EHS); the upregulation of some genes encoding ETC components may represent an adaptive regulatory mechanism for *B. balsamifera* to cope with fluctuations in energy demand and enhance electron transfer efficiency under salt stress (**Figure 7**). In the succinate dehydrogenase/fumarate reductase module associated with Complex Ⅱ, the metabolite fumaric acid was significantly upregulated in the EHS group compared with CK, which is speculated to be related to the adaptive adjustment of metabolic flux between the tricarboxylic acid (TCA) cycle and oxidative phosphorylation pathway under salt stress (**Figure 7**).

### Expression of DAMs and DEGs related to the flavone and flavonol biosynthesis pathway

**Figure 8:**
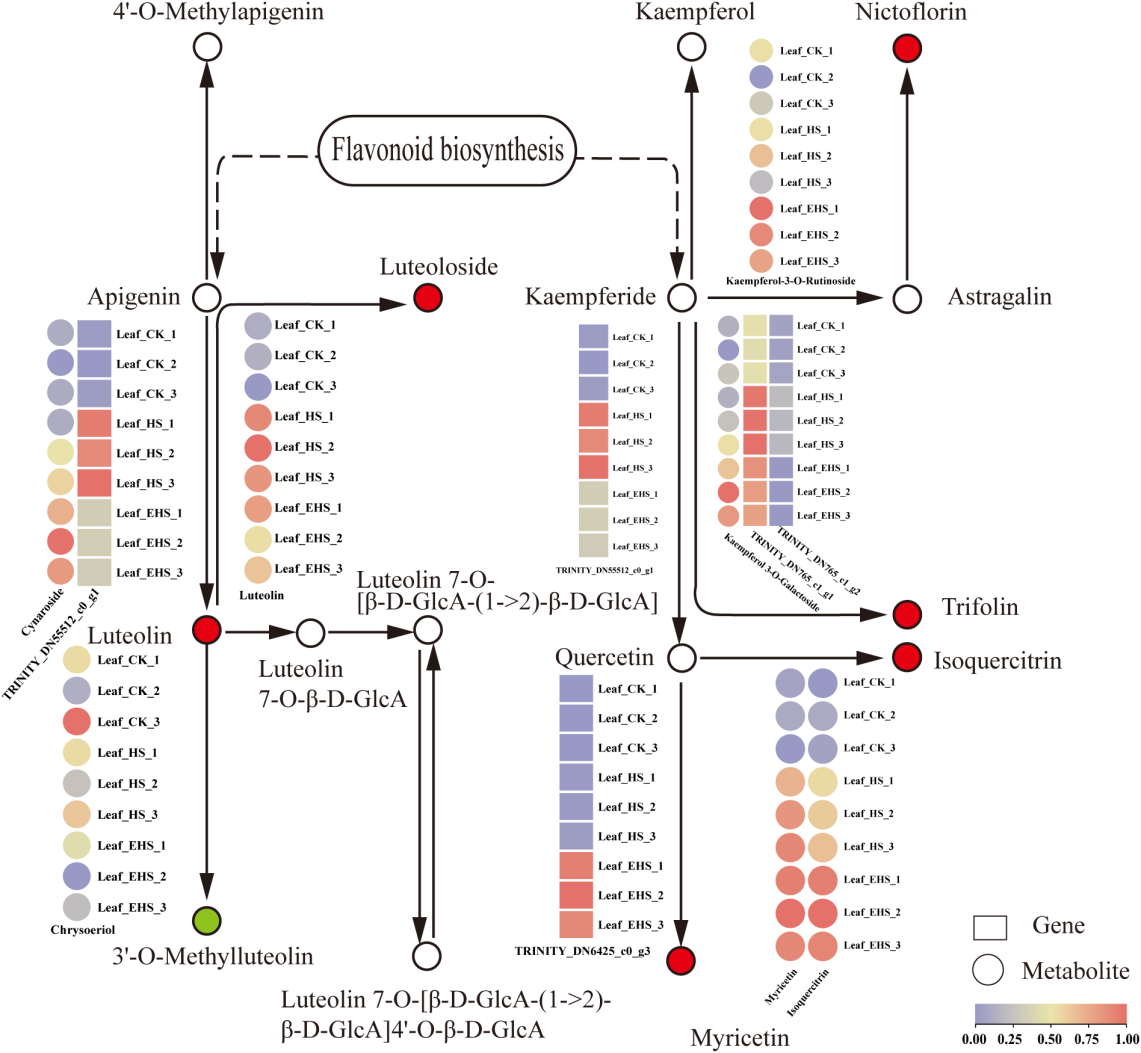
Expression of DAMs and DEGs related to the flavonoid and flavonol biosynthesis pathways. In the schematic diagram of the pathway: boxes represent genes, circles represent metabolites; for the circles corresponding to metabolites, red indicates upregulated metabolites and green indicates downregulated metabolites; the color gradient from purple to red corresponds to the expression levels of the molecules (purple indicates low expression, and red indicates high expression).

Flavones and flavonols are key secondary metabolites for plants in responding to salt stress, exerting functions such as scavenging ROS and maintaining redox homeostasis (Wang et al., 2016). In the flavone and flavonol biosynthesis pathway of *B. balsamifera* under salt stress, at the metabolite level, Chrysoeriol was downregulated, while Kaempferol-3-O-Rutinoside, Luteolin, Cynaroside, Myricetin, and Isoquercitrin were all upregulated; in the metabolic branch from Apigenin to Luteolin, the metabolite Cynaroside was simultaneously upregulated with the TRINITY_DN55512_c0_g1 (Trinity transcript ID, functionally annotated as flavonoid 3’-monooxygenase) (**Figure 8**). As a key catalytic enzyme in this metabolic step, the upregulation of its expression may promote substrate conversion to Luteolin, which is one of the potential driving mechanisms for the enhanced metabolic flux in this branch under salt stress. Meanwhile, TRINITY_DN55512_c0_g1 (flavonoid 3’-monooxygenase) is also involved in the catalysis of Kaempferide to Quercetin, and its expression was significantly upregulated compared with CK under HS and EHS treatments, but slightly lower under EHS than HS (**Figure 8**). This change indicates that the expression of this gene may be subject to moderate feedback regulation under extreme salt stress, so as to maintain the relative homeostasis of pathway metabolic flux.

### Expression of DAMs and DEGs related to the cutin, suberine and wax biosynthesis pathway

**Figure 9:**
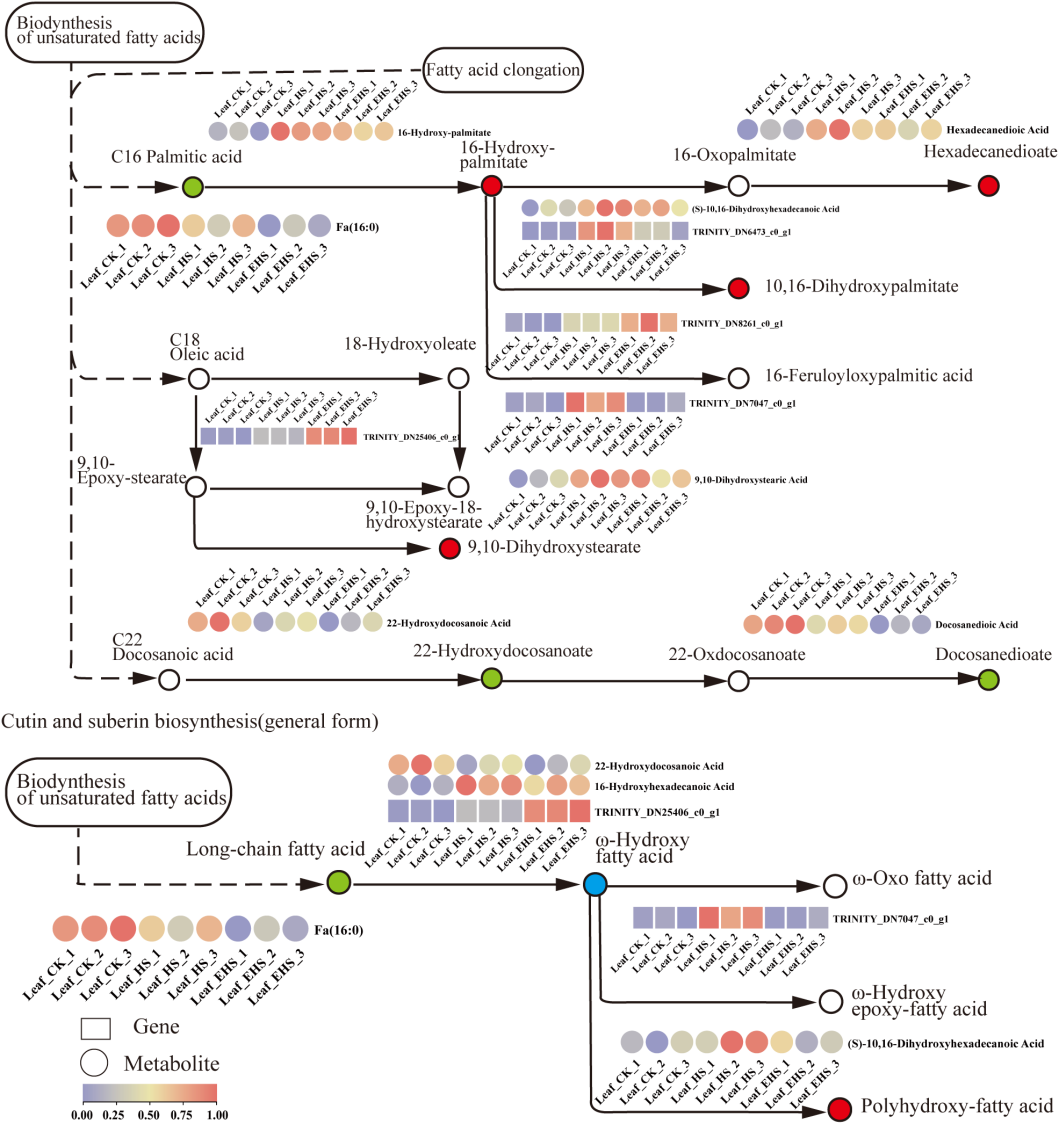
Expression of DAMs and DEGs related to the cutin, cork and wax biosynthesis pathways. In the pathway schematic: red circles represent upregulated metabolites, green circles represent downregulated metabolites, and blue circles represent metabolites that are both upregulated and downregulated; in the heat map region, boxes correspond to genes and circles correspond to metabolites; the color gradient indicates expression levels, with purple representing low expression and red representing high expression.

Cutin, suberine, and wax are key components of the plant epidermal barrier, and the regulation of their biosynthesis constitutes one of the adaptive strategies for *B. balsamifera* in response to salt stress. In the fatty acid elongation and modification branch, C16 palmitic acid was downregulated, while its downstream metabolites (16-Hydroxy-palmitate, Hexadecanedioic Acid, and 9,10-Dihydroxystearic Acid) were significantly upregulated, and 22-Hydroxydocosanoic Acid and Docosanedioic Acid were downregulated—reflecting the differential regulatory patterns of the pathway on the accumulation of fatty acid derivatives with different chain lengths under salt stress. Notably, (S)-10,16-Dihydroxyhexadecanoic Acid was upregulated in both the fatty acid biosynthesis and cutin/suberine biosynthesis pathways; as a cutin monomer precursor, it is speculated that plants synergistically activate the synthesis of this metabolite in both pathways to enhance the structural integrity of the epidermal barrier under salt stress (**Figure 9**). Furthermore, the TRINITY_DN7047_c0_g1 gene (Trinity transcript ID, annotated as peroxygenase-like isoform X2) mediates the conversion of 18-Hydroxyoleate to 9,10-Epoxy-18-hydroxystearate, and its expression was significantly upregulated in HS and EHS groups compared with CK, suggesting a core role in fatty acid modification under salt stress. Meanwhile, genes involved in fatty acid hydroxylation, elongation, and other processes in this pathway were upregulated under salt stress, consistent with the accumulation trends of most of the aforementioned key metabolites—further indicating that the activation of the cutin-suberine-wax biosynthesis pathway is an important physiological regulatory mechanism for in responding to salt stress.

### Validation of RNA-seq data

**Figure 10:**
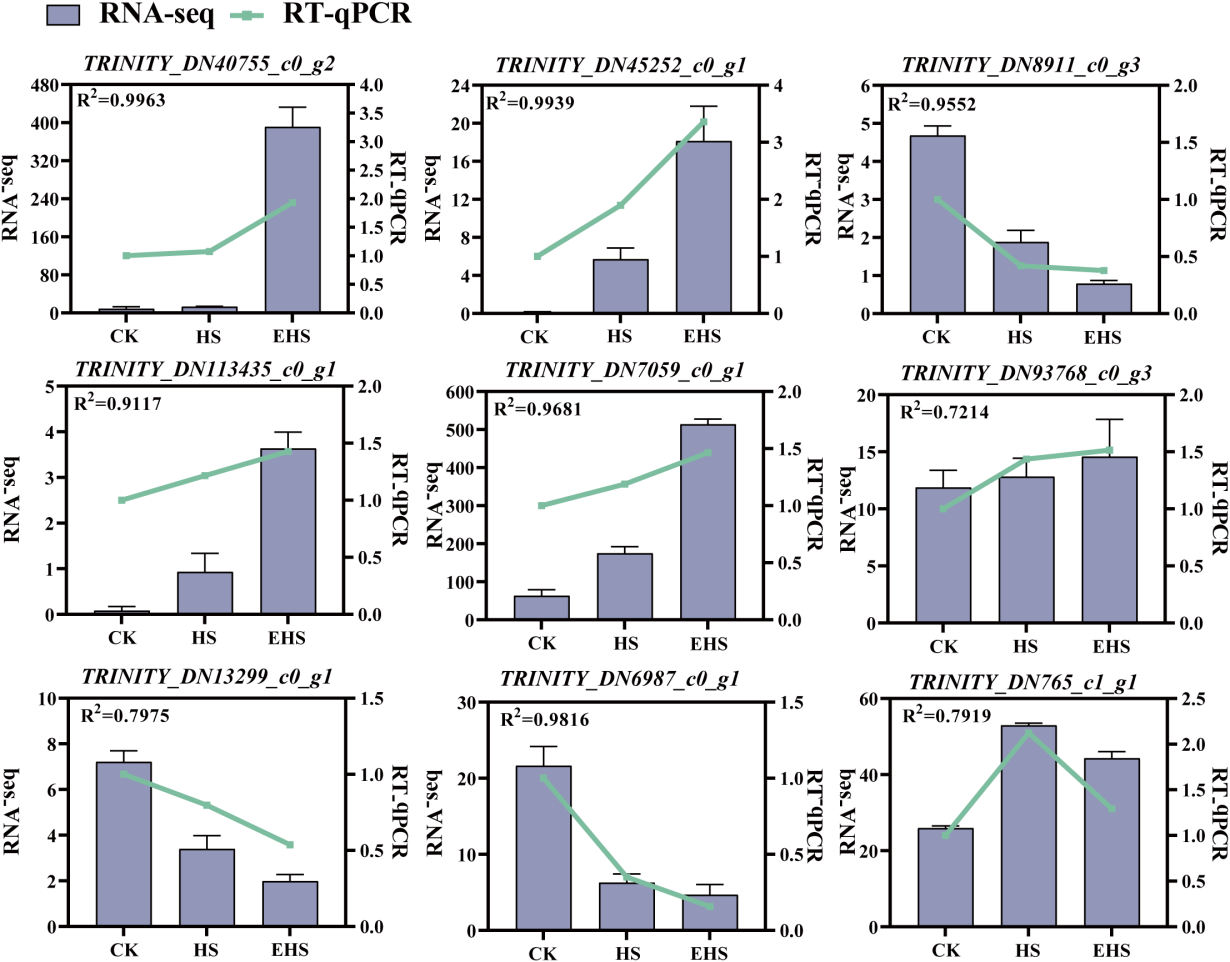
Comparative analysis of RNA-seq and RT-qPCR.

Analysis results showed that changes in the relative expression levels of the 9 selected genes exhibited a highly similar variation pattern to the transcriptomic data (**Figure 10**). This result strongly confirmed the reliability of the transcriptomic analysis data. Furthermore, the differential expression of these genes at the transcriptomic level suggests that they play important roles in the salt tolerance regulation process of *B. balsamifera*.

## Discussion

As one of the major abiotic stresses, salt stress induces cellular dehydration and ionic toxicity that damage cell structures, thereby disrupting plant growth, development, and photosynthetic metabolism (Chaves et al., 2009; Farooq et al., 2017). This study demonstrated that the response of *B. balsamifera* to salt stress is concentration and time-dependent: phenotypically, plants under HS and EHS treatments exhibited wilting and chlorosis at the late stage, while those under CK and LS treatments remained normal, and the inhibition of plant growth was gradually aggravated with the intensification of salt stress. For photosynthetic parameters, the indices under MS, HS and EHS treatments declined progressively with increasing stress severity; the Pn and Tr under EHS treatment were significantly lower than those in the CK group at 24 d, and the Ci in the HS and EHS groups presented a fluctuating trend of decreasing initially and then increasing.This response is consistent with the salt stress response characteristics of *Salix alba* L (Ran et al., 2021), *Cucumis melo* L (Sarabi et al., 2019), *Hordeum vulgare* L (Tavakkoli et al., 2011), and *Helianthus annuus* L., a typical species belonging to the Asteraceae family (Rivelli et al., 2002). Based on the stomatal and non-stomatal limitation theory of photosynthetic inhibition (Xu et al., 2025), it can be inferred that in the early stage of decreased photosynthetic rate in *B. balsamifera*, Ci decreased synchronously with Pn, Tr, and Gs, with stomatal limitation as the dominant factor; after prolonged stress, Ci increased while Pn, Tr, and Gs continued to decline, indicating that non-stomatal limitation became the primary cause.

The physiological adaptation mechanisms of plants in response to salt stress exhibit high conservation, and the activation of antioxidant systems, accumulation of osmoregulatory substances, and remodeling of cell wall structures are core strategies to resist ROS damage and maintain cellular homeostasis (Song et al., 2024). In the present study, the activities of antioxidant enzymes (CAT, POD, SOD) exhibited a pattern of initial increase followed by subsequent decrease under HS and EHS treatments, which is consistent with the response rules reported in *Helianthus annuus.* and *Chrysanthemum morifolium* Ramat., two representative species of the Asteraceae family (Liang et al., 2017; Shahbaz et al., 2025). The variation characteristics of osmotic adjustment substances in *B. balsamifera* are similar to the osmotic regulatory responses of *Cucumis sativus* L. under salt stress (Yu et al., 2025), which constitutes a typical adaptive strategy for plants to sustain cellular osmotic pressure and alleviate dehydration injury through solute accumulation. Notably, the content of LIG increased continuously; by strengthening the cell wall structure to inhibit pathogen invasion, it might alleviate damage caused by excessive ROS accumulation, which is consistent with the stress resistance mechanism of cell wall lignification remodeling in *Solanum lycopersicum* L (Mandal et al., 2011). Furthermore, correlation analysis showed that POD had a strong positive correlation with LIG (r=0.97), reflecting the coordinated regulatory relationship between physiological indices during the stress resistance process.

Under salt stress, the synergistic interaction between metabolic and transcriptional regulation in plants serves as a core molecular mechanism for achieving stress adaptation, and stress intensity is typically a key factor driving differential expression in transcriptomes and metabolomes (Zhang et al., 2021b). Untargeted metabolomics analysis demonstrated that the DAMs identified in both the HS vs CK and EHS vs CK comparisons were significantly enriched in the flavone and flavonol biosynthesis pathway. Within this pathway, DAMs such as Kaempferol-3-O-Rutinoside, Luteolin, and Cynaroside were upregulated, which contribute to salt tolerance responses by enhancing antioxidant capacity—consistent with the stress-related metabolic adaptation mechanisms observed in *Ficus carica* L (Francini et al., 2021). and *Cajanus cajan* L (Song et al., 2022). under salt stress. Transcriptomic analysis revealed that increasing salt concentration may promote transcriptional reprogramming in *B. balsamifera*, and the number of DEGs between the EHS and CK groups was considerably higher than that between the HS and CK groups, implying that plants need to activate a more extensive gene regulatory network to resist adversity under extreme salt stress. Furthermore, DEGs from both the HS vs CK and EHS vs CK comparisons were significantly enriched in the oxidative phosphorylation pathway, which can satisfy the energy requirements of plants under salt stress by remodeling mitochondrial energy metabolism (Farhat et al., 2019).

The synergistic regulation between metabolism and transcription is crucial for plants to precisely respond to differences in stress intensity and achieve gradient adaptation, and the degree of this synergy combined with pathway regulatory specificity collectively determines plant salt tolerance (Li et al., 2024). Furthermore, the absolute values of | rho | obtained from integrated metabolomic-transcriptomic analysis were predominantly distributed in the range of 0.8 to 1.0, indicating a robust synergistic regulatory relationship. The core responsive pathways comprised cutin, suberin and wax biosynthesis, flavone and flavonol biosynthesis, and oxidative phosphorylation. In comparison with other Asteraceae species, the core salt-tolerant pathways in *Taraxacum mongolicum* Hand.-Mazz. under salt stress are featured by a strong correlation between the flavonoid biosynthesis pathway and osmotic adjustment pathways (Liu et al., 2021), while *B. balsamifera* displays a distinct multi-pathway synergistic mode. This species-specific synergistic regulatory network may represent an important molecular foundation underlying its adaptation to high-saline habitats.

Deep analysis of the regulatory mechanisms underlying the core response pathways demonstrated that genes encoding components of the electron transport chain in the oxidative phosphorylation pathway exhibited a significant upregulation trend, which is presumed to optimize energy supply by improving electron transport efficiency, thereby ensuring the basic metabolism of plants under salt stress (Yang et al., 2023). Given the close correlation between the expression of electron transport chain-related genes and energy metabolism, the upregulation of these genes can enhance electron transport efficiency, thus providing adequate energy support for the fundamental photosynthetic carbon assimilation metabolism (e.g., the maintenance of Pn) and physiological and biochemical metabolism (including the synthesis of osmotic adjustment substances and the scavenging of ROS) of *B. balsamifera* (Farhat et al., 2019). Furthermore, fumaric acid (a tricarboxylic acid, TCA cycle intermediate) accumulated significantly in the EHS group, which may result from adaptive changes in the catalytic properties of succinate dehydrogenase; this phenomenon is consistent with the mechanism by which *Glycine max* L. regulates salt tolerance via the TCA cycle (Li et al., 2017), further confirming the conserved role of the TCA cycle in regulating energy metabolism in plants under salt stress.

The flavone and flavonol biosynthesis pathway mediates plant antioxidant defense through the regulation of metabolic flux, constituting an essential metabolic pathway for plant adaptation to environmental stress (Chen et al., 2023). Under salt stress, in the metabolic branch from apigenin to luteolin in *B. balsamifera*, the metabolite cynaroside and the gene TRINITY_DN55512_c0_g1 (Trinity transcript ID, with functional annotation of flavonoid 3’-monooxygenase) were synchronously upregulated; integrating the documented findings that salt stress can induce cynaroside accumulation (Genzel et al., 2021) and that the flavonoid 3’-monooxygenase gene participates in plant stress responses (Awasthi et al., 2016), it is inferred that this gene acts as the key driver for the elevated flux of this metabolic branch. Additionally, this gene is also involved in the catalysis of Kaempferide to Quercetin; its expression was significantly upregulated under both HS and EHS treatments, but slightly lower under EHS than HS. Considering the requirement for metabolic flux homeostasis under extreme salt stress, it is speculated that the expression of this gene is subject to moderate feedback regulation to avoid excessive accumulation or depletion of pathway metabolites (Zhou et al., 2025). Further analysis revealed that DAMs such as Kaempferol-3-O-Rutinoside, Luteolin, Myricetin, and Isoquercitrin were upregulated; based on the antioxidant properties of flavonoids, these metabolites are inferred to synergistically enhance ROS scavenging capacity with SOD, CAT, and POD, thereby reducing membrane damage caused by MDA accumulation.

Strengthening of the epidermal barrier is a key strategy for plants to reduce water loss and enhance structural stability under salt stress, and its regulation is closely associated with plant water status and cell wall properties (Moog et al., 2023; Zhou et al., 2025). Under salt stress, the cutin, suberine, and wax biosynthesis pathway of *B. balsamifera* exerts differential regulation on fatty acid derivatives with different chain lengths: C16 palmitic acid was downregulated, while its downstream metabolites, such as 16-Hydroxy-palmitate, Hexadecanedioic Acid, and 9,10-Dihydroxystearic Acid were significantly upregulated. Given the role of fatty acid derivatives as precursors for cutin and suberine, it is inferred that this regulation reduces water loss by optimizing the epidermal barrier. The cutin monomer precursor (S)-10,16-Dihydroxyhexadecanoic Acid was significantly upregulated in both the fatty acid biosynthesis and cutin/suberine biosynthesis pathways; considering its core role in cuticle formation, its synergistic synthesis is speculated to enhance epidermal barrier integrity for stress adaptation. Additionally, the TRINITY_DN7047_c0_g1 gene (Trinity transcript ID, annotated as peroxygenase-like isoform X2) was significantly upregulated in HS and EHS groups, which is speculated to be a core gene for fatty acid modification under salt stress. The upregulation of genes in this pathway was synergistic with the increase in LIG content, and the two are likely to collectively participate in strengthening the plant cell wall, thereby improving water retention capacity and stress resistance of *B. balsamifera* (Martínez et al., 2017).

These results demonstrate that the response of *B. balsamifera* to salt stress is concentration and time-dependent, achieving synergistic adaptation through the coordinated regulation of phenotypic, photosynthetic, physiological, biochemical, metabolic, and transcriptional remodeling. Oxidative phosphorylation, Flavone and flavonol biosynthesis, and Cutin, suberine and wax biosynthesis are the core conserved pathways underlying its salt stress response. The biosynthesis of medicinal components such as blumeatin is closely correlated with relevant metabolic pathways, and this study demonstrates that the activation of the flavone and flavonol biosynthesis pathway under salt stress not only participates in the salt-tolerant defense of *B. balsamifera*, but may also regulate the accumulation of its medicinal components. Nevertheless, the present study has not validated the functions of key salt tolerance-related genes and differentially accumulated metabolites, nor explored the mechanism of ion homeostasis in *B. balsamifera* under salt stress. In subsequent research, gene editing technologies can be utilized to verify the functions of core candidate genes, and ionomics can be integrated to dissect the regulatory mechanism of ion balance in response to salt stress.

## Conclusion

This study investigated the salt tolerance mechanism of *B. balsamifera* by integrating phenotypic, photosynthetic, physiological, and biochemical analyses with metabolomics and transcriptomics. Under salt stress, the photosynthetic process shifted from stomatal limitation to non-stomatal limitation with increasing stress intensity; at the physiological level, *B. balsamifera* scavenged ROS via SOD, CAT, and POD, activated the osmotic adjustment system, and enhanced cell wall rigidity by increasing LIG content to resist salt damage. Metabolomic analysis identified 677 and 692 DAMs under HS and EHS stresses, respectively, with both groups showing significant enrichment in the flavone and flavonol biosynthesis pathway. Transcriptomic analysis revealed 30,213 and 13,644 DEGs under HS and EHS stresses, respectively, with common enrichment in the oxidative phosphorylation pathway. Integrated multi-omics analysis further demonstrated that oxidative phosphorylation, flavone and flavonol biosynthesis, and cutin-suberine-wax biosynthesis are the core pathways mediating the salt stress response of *B. balsamifera*, which synergistically copes with salt stress by regulating DAMs such as fumaric acid, Kaempferol-3-O-Rutinoside, and Luteolin, as well as DEGs including flavonoid 3’-monooxygenase and peroxygenase-like isoform X2. This study systematically elucidates the salt tolerance mechanism of *B. balsamifera*, providing a scientific basis for its salt-tolerant breeding practices and the development of medicinal plant resources on salinized marginal lands.

## Data Availability

The datasets presented in this study can be found in online repositories. The names of the repository/repositories and accession number(s) can be found in the article/**Supplementary Material**.

## Supplementary material

Supplementary Table 1 Genes and corresponding primers used in the qPCR test.

Supplementary Table 2 Flavone and flavonol biosynthesis pathway related DAMs.

Supplementary Table 3 Quality control statistics of sequencing data.

Supplementary Table 4 Comparison of sequencing results with assembly results.
